# Informed consent and informed intervention: SARS-CoV-2 vaccinations not just call for disclosure of newly emerging safety data but also for hypothesis generation and testing

**DOI:** 10.1186/s40001-021-00558-y

**Published:** 2021-08-06

**Authors:** Johannes C. Fischer, Albrecht G. Schmidt, Edwin Bölke, Verena Keitel, Torsten Feldt, Björn Jensen, Noemi F. Freise, Dieter Häussinger, E. Marion Schneider, Derik Hermsen, Detlef Kindgen-Milles, Wolfram Trudo Knoefel, Jan Haussmann, Balint Tamaskovics, Christian Plettenberg, Kathrin Scheckenbach, Stefanie Corradini, Jutta Rox, Vera Balz, Kitti Maas, Livia Schmidt, Olaf Grebe, Anja Erhardt, Peter Arne Gerber, Matthias Peiper, Bettina Alexandra Buhren, Artur Lichtenberg, Amir Rezazadeh, Wilfried Budach, Christiane Matuschek

**Affiliations:** 1 Institute for Transplantation Diagnostics and Cell Therapeutics, Medical Faculty, University Hospital Dusseldorf, Heinrich-Heine-University, 40225 Düsseldorf, Germany; 2 Department of Radiation Oncology, Medical Faculty, University Hospital Dusseldorf, Heinrich-Heine-University Dusseldorf, Moorenstr. 5, 40225 Düsseldorf, Germany; 3 Department of Gastroenterology, Hepatology and Infectious Diseases, Medical Faculty, University Hospital Dusseldorf, Heinrich-Heine-University Dusseldorf, Moorenstr. 5, 40225 Düsseldorf, Germany; 4 Division of Experimental Anesthesiology, University Hospital Ulm, Ulm, Germany; 5 Central Institute for Laboratory Diagnostics and Clinical Chemistry, Medical Faculty, Heinrich-Heine University, Düsseldorf, Germany; 6 Department of Anesthesiology, Medical Faculty, Heinrich Heine University, Dusseldorf, Germany; 7 Department of Surgery and Interdisciplinary Surgical Intensive Care Unit, Medical Faculty, Heinrich Heine University, Düsseldorf, Germany; 8 Medicine, Department cBITE, Maastricht, The Netherlands; 9 Department of Otolaryngology–Head and Neck Surgery, Medical Faculty, Heinrich Heine University, Düsseldorf, Germany; 10 Department of Radiation Oncology, University Hospital, LMU Munich, Munich, Germany; 11 Department of Cardiology and Rhythmology, Petrus Hospital, Wuppertal, Germany; 12 Institute of Virology, University of Witten/Herdecke, Witten, Germany; 13 Medical Faculty, University of Dusseldorf, Düsseldorf, Germany; 14 Department of Cardiac Surgery, Medical Faculty, University of Dusseldorf, Düsseldorf, Germany

## Abstract

**Background:**

COVID-19 infection is a major threat to patients and health care providers around the world. One solution is the vaccination against SARS-CoV-2.

**Methods:**

We performed a comprehensive query of the latest publications on the prevention of viral infections including the recent vaccination program and its side effects.

**Results:**

The situation is evolving rapidly and there is no reasonable alternative to population-scale vaccination programs as currently enrolled.

**Conclusion:**

Therefore, regulatory authorities should consider supplementing their conventional mandate of post-approval pharmacovigilance, which is based on the collection, assessment, and regulatory response to emerging safety findings.

On April 09, 2021, the Pharmacovigilance Risk Assessment Committee (PRAC) of the European Medicines Agency (EMA) announced that it has initiated a safety signal review to assess reports of capillary leak syndrome in people vaccinated with Vaxzevria (previously COVID-19 Vaccine AstraZeneca) [https://www.ema.europa.eu/en/news/meeting-highlights-pharmacovigilance-risk-assessment-committee-prac-6-9-april-2021]. According to PRAC’s announcement, “five cases of this very rare disorder, characterized by leakage of fluid from blood vessels causing tissue swelling and a drop in blood pressure were reported in the EudraVigilance database”. At this stage, it is not yet clear whether there is a causal association between vaccination and the reports of capillary leak syndrome. These reports point to a ‘safety signal’—information about new or changed adverse events that may be associated with a drug and warrant further investigation. PRAC will evaluate all available data to decide if a causal relationship can be confirmed or not. In cases where a causal relationship is confirmed or considered likely, regulatory action is necessary to minimize the risk. This usually takes the form of updating the summary of product characteristics and “package leaflet.”

This recent announcement by EMA illustrates the challenge posed by the current rollout of population-wide vaccination programs to fight the ongoing, devastating COVID-19 pandemic. This population-wide rollout is based on data derived from developmental studies to vaccinate tens of thousands of patients. These patient numbers are at least three orders of magnitude lower than the tens and hundreds of millions of citizens who are now vaccinated. The only remedy available to address the intrinsic dilemma of discrepancy of scale is the instant collection, assessment and communication of pharmacovigilance data. Whatever the outcome of the further investigations in regard to the potential association of systemic capillary leak syndrome (SCLS, also known as Clarkson disease [1]) with DNA vector vaccines will be, it is apparent that the PRAC has lived up to this first level of challenge of instant data disclosure.

Unfortunately, the EMA announcement does little to address the next level of challenge, now facing thousands of vaccinating physicians and millions of citizens: How should this information be interpreted? Is this just an ice floe, or is it an iceberg which should affect decisions? And if so, in what way? The COVID-19 outbreak in Wuhan, China hit European countries unprepared, therefore this next level of interpretation has largely been left to politicians in charge of societies’ healthcare systems as well as their expert advisors. In the absence of guiding evidence, politicians took resort with trusted experts who volunteered their levels of understanding, often presented with great authority to media and public. In not too few instances, advisory inputs were advanced into political interventions directly and without further parliamentary debate, justified by the urgency of need, inevitably leaving questions regarding potential alternatives unanswered. This commentary submits that this practice, even though customary by now, deserves careful re-consideration in the current vaccination phase of the pandemic. This phase requires the voluntary informed consent of informed citizens and will predictably be difficult to master (or with substantive societal cost only) without.

In a scientific setting, no firm conclusions must be drawn in the absence of conclusive evidence. Nevertheless, any newly emerging data evidence will and should be inquired for plausible hypotheses to explain unexpected findings. The deriving exploratory hypotheses will then become tested in confirmatory fashions for evidence-based conclusive assessment. In the present situation, rare and very rare phenomena were observed with a DNA vector vaccine yet not with the use of mRNA vaccines. It is therefore not too early to formulate the hypothesis, that the rare and very rare occurrence of thrombotic-thrombopenic events and capillary leak syndrome may be related to either the encoded transcripts, the DNA nature, or the viral vector nature of DNA vector vaccines. With this exploratory hypothesis as a starting point, it becomes intuitive to inquire whether the greater stability and the wider dissemination of DNA vector vaccines, not categorically excluding infection of endothelial cells, may contribute to rare yet potentially severe safety events involving endothelial linings. With a respective preliminary hypothesis, it becomes possible to search for further evidence informing the hypotheses itselves and specifying them for conclusive experimental testing. In the case of thrombotic-thrombopenic events, the documented presence of a so-called RGD motif within the S protein (the dominant viral antigen employed for vaccination) [2] with the principle capacity of binding to integrins expressed on platelets and endothelial cells requires active consideration [3]. Of note, the putative binding of platelets can be subjected to enable experimental testing. Similarly, the reported occurrence of otherwise exceptionally rare SCLS in a COVID-19 patient suffering from monoclonal gammopathy invites for the generation of additional hypotheses, as the viral nature of the intervention was implicated in the absence of adequate endothelial shielding immunoglobulin levels [4].

This comment is submitted because direct follow-up by focused hypothesis-driven experiments may firstly provide better insight into the pathomechanisms of unexpected events and their association with the intervention. Second, this approach may lead to a more precise and informed risk stratification (which, at best, will allow informed risk/benefit assessments at the individual patient level) and a better targeting of interventions. Last but not least, a better pathomechanistic understanding may enable the early detection of safety signals for the respective vaccines and vaccine candidates of the class.

The current situation is evolving rapidly, and there is no reasonable alternative to population-wide vaccination programs as currently rolled out. Therefore, regulatory authorities should consider supplementing their conventional mandate of post-approval pharmacovigilance, which is based on the collection, assessment, and regulatory response to emerging safety findings. In the given situation, they may also consider the generation of evidence-based, biologically plausible hypotheses to be tested with the involvement of manufacturer resources and public science. In the past year, vaccine developments have been achieved at unprecedented pace—in the now ongoing vaccination phase an even more impressive societal gain may become apparent: Best-informed voluntary consent of millions of citizens receiving highly innovative interventions assessed and administered by best-informed and best-informing healthcare professionals.

## Data Availability

All data and materials can be accessed via CM and FM.
